# High Value-Added Application of Two Renewable Sources as Healthy Food: The Nutritional Properties, Chemical Compositions, Antioxidant, and Antiinflammatory Activities of the Stalks of *Rheum officinale* Baill. and *Rheum tanguticum* Maxim. ex Regel

**DOI:** 10.3389/fnut.2021.770264

**Published:** 2022-01-24

**Authors:** Li-Xia Dai, Xiaolou Miao, Xiao-Rong Yang, Li-Ping Zuo, Zhi-Hui Lan, Bing Li, Xiao-Fei Shang, Feng-Yuan Yan, Xiao Guo, Yu Wang, Ji-Yu Zhang

**Affiliations:** ^1^Key Laboratory of New Animal Drug Project, Key Laboratory of Veterinary Pharmaceutical Development of Ministry of Agriculture, Lanzhou Institute of Husbandry and Pharmaceutical Sciences, Chinese Academy of Agricultural Sciences, Lanzhou, China; ^2^The First People's Hospital of Lanzhou City, Lanzhou, China; ^3^State Key Laboratory of Tibetan Medicine Research and Development, Qinghai University, Xining, China

**Keywords:** *Rheum officinale*, *Rheum tanguticum*, nutritional value, chemical compositions, antioxidant activity

## Abstract

Rhubarb plants (*Rheum officinale* and *R. tanguticum*) have edible stalks. In this work, we aimed to compare the nutritional properties, chemical compositions, and bioactivities of *R. officinale* (SRO) and *R. tanguticum* (SRT) stalks and to analyze the composition–function relationship. Results showed that the two stalks were good sources of fiber, as well as minerals. They contained abundant essential amino acids and essential fatty acids to regulate the immunity and prevent some chronic diseases; the contents of polyunsaturated fatty acids were 2,244.32 mg/100 g and 2,844.69 mg/100 g, respectively. The antioxidant activity were also proved. Metabolomics showed that SRO and SRT contained abundant phenolic acids. Due to the higher concentrations of flavones, SRT has better antiinflammatory activities than SRO by inhibiting NF-κB signaling pathway. Rhubarb stalks exhibited good safety in acute toxicity and cytotoxicity tests. This work indicated that the two stalks have nutritional value, safety, and bioactivities, and could be used as sources of nutritional ingredients for regulating the immunity of body in food industry.

## Introduction

The genus *Rheum* (Polygonaceae) contains ~50 species of perennial herbaceous plants, which are widely distributed and cultivated worldwide (1). The dried rhizomes and roots of *Rheum palmatum, Rheum tanguticum*, and *Rheum officinale* have been prescribed as official rhubarbs and are listed in many pharmacopeias, such as the Chinese Pharmacopeia (2), the United States Pharmacopeia (3), and the European Pharmacopeia (4), with a wide range of pharmacological activities (5). In the USA, Europe, and the Middle East, the stalks of some species are usually consumed as fruit instead of vegetables, including *R. ribes* and *R. rhabarbarum* (6–8), which possess valuable natural active ingredients and dietary fiber. *R. rhabarbarum* is primarily utilized for culinary purposes to prepare various dishes, such as desserts, cakes, mousses, juices, wines, and fruit teas (9).

As two important herbs in this genus, *R. officinale* and *R. tanguticum* are widely employed as folk medicines in some Asian countries (10). In China, these two species are cultivated in western regions, such as Gansu, Qinghai, Sichuan, Shanxi, and Guizhou provinces (10, 11), and approximately 7,000 tons of these two species are produced every year. Phytochemical research showed that the dried rhizomes and roots of the two species were rich in active compounds, such as anthraquinones, flavonoids, stilbenes, and tannins (5, 12, 13). These plants are widely employed to treat constipation, abdominal pain, diarrhea, jaundice, and inflammation of some organs, as they are believed to possess antiviral, antiinflammatory, antimicrobial, antioxidant, and antitumor activities (5). Due to the sour taste of these plants, the stalks of the two species were eaten as vegetables by local people and animals in some regions, especially in May and June of each year. However, the nutrients of the aerial parts of rhubarb have not been comprehensively investigated, and the chemical composition and pharmacological activities of these plants have not been elucidated.

In this work, the nutritional values of the stalks of *R. officinale* and *R. tanguticum* stalks were compared and evaluated firstly, and their acute toxicities and cytotoxicity were studied. Then, their chemical compositions were analyzed by LC–MS-based metabolomics. The antioxidant and antiinflammatory activities, their potential mechanism of actions, and the structure–function relationship analysis of these two stalks were investigated. This work may help to demonstrate the potential health functionality and nutraceutical applications of the stalks and may help to establish the foundation for future research attempting to develop the two species as food ingredients.

## Materials and Methods

### *R. officinale* and *R. tanguticum*

Stalks of *Rheum officinale* (SRO) and *Rheum tanguticum* (SRT) were obtained from Kang County of Gansu Province, China, in June 2020. These samples were identified by Professor Chaoying Luo from the Lanzhou Institute of Husbandry and Pharmaceutical Sciencs, CAAS. The voucher specimens were submitted to the herbarium of the Lanzhou Institute of Husbandry and Pharmaceutical Sciences, CAAS (Lanzhou, China). The fresh stalks of two samples were dried under shadow for use in biochemical investigation.

### Nutritional Value of *R. officinale* (SRO) and R. tanguticum (SRT) Stalks

#### Proximate Compositions

The proximate compositions of SRO and SRT stalks were analyzed according to a previously described method (14). Briefly, the contents of crude lipids in two species were determined with a Soxtec 2050 Automatic Soxhlet extraction analyzer (FOSS, Denmark). The total protein contents were obtained by determining the nitrogen content of the two species using a Kjeltec 8200 nitrogen analyzer (FOSS, Denmark). The total dietary fiber in SRO and SRT was measured by the Prosky-AOAC method using a TDF-100A enzymatic kit (Sigma, USA). Finally, the moisture and ash of two species were measured according to the previously described methods (15, 16). Three replicates were employed.

#### Mineral Compositions

The mineral compositions of SRO and SRT were determined according to the previously described AOAC methods 984.27 and 985.01 (modified) (14). First, after digestion with HCl and water, the samples were filtered and transferred to flasks, and the mineral compositions were subsequently determined using inductively coupled plasma mass spectrometry (Agilent 7900, USA). The absorption wavelengths were set at 196.0 nm for selenium (Se), 213.6 nm for phosphorus (P), 213.8 nm for zinc (Zn), 248.3 nm for iron (Fe), 279.5 nm for manganese (Mn), 285.2 nm for magnesium (Mg), 324.8 nm for copper (Cu), 422.6 nm for calcium (Ca), 589.6 nm for sodium (Na), and 766.5 nm for potassium (K). The contents of each mineral are presented as mg/100 g, and the contents of each mineral were calculated according to the analytic curves of standards. Three replicates were employed.

#### Amino Acids Compositions

Amino acid compositions of SRO and SRT were determined according to a previously described method (17). The samples were digested with 10 mL of 6 M HCl in sealed ampoule at 110°C for 24 h. Hydrolyzed sample was filtered, and the supernatant was transferred to a bottle to dry at 60°C. Then 0.2 M sodium citrate buffer of pH 2.2 was utilized to dissolve the samples, and then they were centrifuged. The supernatant was obtained and subsequently subjected to a Biochrom 30^+^ Automatic Amino Acid Analyzer (Biochrom, England) with an ion-exchange column to determine the amino acid composition. The absorption wavelengths were set at 440 nm for proline and 570 nm for the other amino acids. The standard solution of amino acids (Sigma, USA) was prepared at a concentration of 100 nmol/mL.

#### Fatty Acids Compositions

The fatty acid compositions of the samples were determined according to a previously described method (18). First, the dried samples of SRO and SRT (20 mg) were mixed with 2.0 mL of an internal standard (undecanoic acid, Sigma, USA) methanol solution and 100 mg pyrogallol in a glass tube. They were heated at 80°C for 1 h, and then 15% boron trifluoride in methanol (7 mL) was added into the tube to convert fatty acids to esters at 80°C for 1 h again. When the solutions were cooled, 15 mL of hexane and saturated sodium chloride solution were added, and the hexane layer was extracted and transferred into a vial. The derivatized fatty acids were separated using an Agilent HP-88 column (60 × 0.25 mm, 0.2 μm) and determined by autosampler gas chromatography (GC) with a flame ionization detector. The temperatures were set at 270°C for the injector and 280°C for the detector. The oven temperature conditions were set to 100°C and held for 13 min, increased to 180°C (10°C/min) and held for 6 min, subsequently raised to 200°C (1°C/min), held for 20 min, and then finally increased to 230°C (4°C/min) and held for 10.5 min. Helium was employed as the carrier gas, and the split ratio was 100:1. The fatty acid methyl ester was identified by comparing the GC retention times with those of a mixture of their standards (Sigma, USA), and fatty acids were calculated using fatty acid conversion factors from its methyl ester [AOAC Official Method 996.06 (modified)] (14).

### UHPLC–QQQ–MS-Based Metabolomics

*Rheum officinale* and SRT samples were dried and crushed using a freeze dryer (Scientz-100F, Ningbo) and a mixer mill (MM 400, Retsch, Germany), respectively. After extracting using 1.2 mL of 70% methanol for 12 h at 4°C, the samples were centrifuged at 12,000 × g for 10 min and were filtered by microporous membrane (0.22 μm pore size; ANPEL, Shanghai, China). Three replicates were employed.

Subsequently, the samples were analyzed using an UPLC-ESI-MS/MS system (UPLC, SHIMADZU Nexera X2; MS, Applied Biosystems 4500 Q TRAP). An Agilent SB C_18_ column (1.8 μm, 2.1 × 100 mm) was utilized for separating the samples. The solvent system was composed of 0.1% acetic acid solution (A) and acetonitrile with 0.1% acetic acid (B), and a gradient elution method was applied as follows: 0–9 min 5–95% B; 9–10 min 95% B, 11–11.1 min 95–5% B; 11.1–14 min 5% B; the flow rate was 0.35 mL/min. The temperature was set to 40°C, and the injection volume was 4 μL.

Linear ion trap scans (LIT) and triple quadrupole (QQQ) scans were acquired on a triple quadrupole-linear ion trap mass spectrometer (Q TRAP), AB4500 Q TRAP ultra performance liquid chromatography (UPLC)/MS/MS System, equipped with an ESI Turbo Ion-Spray interface, operating in a positive and negative ion mode and controlled by Analyst 1.6.3 software (AB Sciex). The operating parameters of the ESI source were as follows: ion source, turbo spray; source temperature, 550°C; ion-spray voltage, 5500 V; ion source gas I, gas II, and curtain gas, 30 psi. QQQ scans were acquired in multiple reaction monitoring (MRM) mode with the collision gas (nitrogen) set to 5 psi. The instrument tuning and mass calibration were performed with 10 and 100 μmol/L polypropylene glycol solutions in the QQQ and LIT modes, respectively. A specific set of MRM transitions was monitored for each period in accordance with the metabolites eluting (19, 20).

### Toxicity

#### Acute Toxicity

This test was approved by the Institute Animal Care and Use Committee of Lanzhou Institute of Husbandry and Pharmaceutical Sciences, CAAS (SYXK-2014-0002). Mice (18–22 g) were obtained from the Lanzhou Veterinary Institute and were randomly divided into groups (*n* = 10). After being ground to 200 mesh, SRO and SRT were dissolved in distilled water to concentrations of 2,000–5,000 mg/kg and administered orally to mice. Finally, the mice were observed continuously for behavioral changes for the first 4 h and were subsequently observed for mortality for 24 h after administration.

#### Cytotoxicity

The popular murine macrophage cell line, RAW 264.7, is often used to initially screen natural products for bioactivity and to predict their potential effect, especially for inflammatory and oxidant response (21). It also plays an important role in inflammatory processes and release various cytokines. This cell line has been widely used to investigate the antiinflammatory and antioxidant activity (22, 23); hence, we adopted this cell line. RAW 264.7 cells, obtained from Prof. Zhang's lab., Lanzhou Institute of Husbandry and Pharmaceutical Sciences, CAAS (Lanzhou, China), was cultured in culture medium prepared with 10% FBS and 90% DMEM under a humidified incubator of 5% CO_2_ at 37°C. The cytotoxicity of SRO and SRT against RAW 264.7 cells were evaluated using the ZETA cell counting kit (ZETA Life, U.S.A.). Detailed, RAW 264.7 cells at a density of 1 × 10^5^ cells/well (100 μL) were incubated in 96-well-plates for 24 h, and 10 μL of the SRO and SRT (25–200 μg/mL) were added to each well and incubated again for 24 h. CCK-8 agent (10 μL) were added and cultured continuously for 30 min, and the absorbance was measured at 450 nm using a Multiskan Go Microplate Spectrophotometer (Thermo Scientific., U.S.A). DMSO (0.1%) was used as a control, and three replicates were performed.

### Antioxidant Activity

#### *In vitro* Antioxidant Activity

##### DPPH Radical Scavenging Activity Assay

The DPPH radical scavenging activities of SRO and SRT were evaluated (24). Briefly, 100 μL of SRO and SRT (50–1,000 μg/mL) were added into 96-well-plates and mixed with DPPH (100 μL, 0.2 mM) in methanol solution at 37°C for 30 min in the dark. The samples were measured using a microplate spectrophotometer at 517 nm. Distilled water and ascorbic acid (Vc) were used as a negative control and positive control, respectively. Three replicates were employed.

##### Hydroxyl Radical Scavenging Activity Assay

The hydroxyl radical scavenging activities of SRO and SRT were evaluated (24). Fifty microliters of SRO and SRT (50–1,000 μg/mL) were mixed with 50 μL of FeSO_4_ (9 mM), 50 μL of ethanol-salicylate (9 mM), and H_2_O_2_ (3.8 mM) were added to 96-well-plates and incubated at 37°C for 30 min. The samples were measured using a microplate spectrophotometer at 510 nm. Distilled water was utilized as a negative control, and Vc (20–600 μg/mL) was utilized as a positive control for comparison. Three replicates were employed.

##### Superoxide Radical Scavenging Activity Assay

The superoxide radical scavenging activities of SRO and SRT were evaluated according to a previously described method (24). Fifty microliters of SRO and SRT (50–1,000 μg/mL) were mixed with 100 μL of NADH-2Na (557 μM), 50 μL of PMS (45 μM), and 50 μL of NBT (108 μM) and incubated at 25°C for 5 min. The samples were analyzed using a microplate spectrophotometer at 510 nm. Distilled water was utilized as a negative control, and Vc (20–100 μg/mL) was utilized as a positive control for comparison. Three replicates were performed.

#### Cellular Antioxidant Activity

For evaluating the *in vivo* antioxidant activities of SRO and SRT, the SOD (superoxide dismutase) and CAT (catalase) activities and MDA (malondialdehyde) content of the RAW 264.7 cells were measured. Briefly, RAW 264.7 cells (2 mL) at a density of 2 × 10^5^ cells/well were incubated in 6-well-plates for 24 h. The cell supernatants were discarded, and then 2 mL of the SRO and SRT (25–250 μg/mL), or the positive control Vc (10 μg/mL) were acceded and cultured for 1 h. Finally, 6 mM hydrogen peroxide (H_2_O_2_) was used to stimulate RAW 264.7 cells for 24 h, the cells were collected to determine the SOD and CAT activities and MDA content using the relative assay kits (Solarbio, China). For SOD, it was measured by using nitro blue tetrazolium as a substrate. After adding reagents, according to the kit, to supernatant and then incubating at 37°C for 40 min, color developing agent was added and kept for 10 min, and the absorbance was measured at 560 nm. For CAT, it was determined by using H_2_O_2_ as a substrate with CAT assay kit, and the absorbance was scanned at 240 nm. The concentration of MDA was measured at 450, 532, and 600 nm. Three replicates were performed.

### Antiinflammatory Assay

The antiinflammatory activities of SRO and SRT were investigated by determining the production of three inflammatory cytokines, nitric oxide (NO), interleukin-1β (IL-1β), and tumor necrosis factor-α (TNF-α) of RAW 264.7 cells induced by lipopolysaccharide (LPS). RAW 264.7 cells (500 μL) at a density of 1 × 10^5^ cells/well were incubated in 48-well plates for 24 h, and the cell supernatants were discarded. Then, 500 μL of the SRO and SRT (25–250 μg/mL) were acceded and cultured for 1 h; dexamethasone (DEX) (10 μg/mL) was used as a positive control. Afterwards, LPS (1 μg/mL) was added to each well, and RAW 264.7 cells were stimulated for 24 h to cause inflammation. The cells were collected to determine the levels of NO, IL-1β, and TNF-α using enzyme-linked immunosorbent assay kits from Nanjingjiancheng Bio (NJJCBIO, China). The concentrations were calculated by the different standard curve equations, and the absorbance was measured at a wavelength of 450 nm. Three replicates were performed.

### Measurement of NF-κB p65 Content

RAW 264.7 cells (2 mL) at a density of 2 × 10^5^ cells/well were incubated in 6-well-plates for 24 h. The cell supernatants were discarded, and then 1 mL of the SRO and SRT (10–100 μg/mL) were acceded and cultured for 1 h. LPS (1 μg/mL) was added to each well for stimulating 24 h to cause inflammation. Finally, the cells were collected to determine the levels of nuclear factor kappa-B (NF-κB) p65 using NF-kB p65 Total SimpleStep ELISA kit (ab176648) (Abcam, China), and the protein concentration was determined using BCA kit (Solarbio, China).

### Western Blot Analysis

RAW 264.7 cells (2 mL) at a density of 2 × 10^5^ cells/well were incubated in 6-well-plates for 24 h. The cell supernatants were discarded, and then 2 mL of the SRO and SRT (10–100 μg/mL) were acceded and cultured for 1 h. LPS (1 μg/mL) was used to stimulate RAW 264.7 cells for 24 h, the cells were collected. The protein was separated on SDS-PAGE and transferred to PVDF membranes, which were blocked in 5% bovine serum albumin for 1 h at room temperature and incubated overnight. Immunoblotting was carried out with the following primary antibodies, including anti-nuclear factor-αB (IκB) alpha (ab32518), anti-IαB alpha (phospho S36) (ab133462), anti-NF-κB p65 (ab32536), and anti-NF-αB p65 (phospho S536) (ab76302) from Abcam (Shanghai, China). Relative protein expression was quantified compared with β-actin level.

### Measurement of the Cellular Reactive Oxygen Species Production

The level of intracellular ROS production in RAW264.7 cells was determined using 2′,7′-dichlorodihydrofluorescein diacetate (DCFH-DA) (NJJCBIO, China). After 24 h of treatment with LPS (1 μg/mL), cells were incubated with DCFH-DA (2 mM) at 37°C for 30 min in darkness and washed with PBS. Finally, the samples were photographed (excitation/emission 500/525 nm) using a LSM-800 with Airyscan (Carl Zeiss Microscopy GmbH, German).

### Measurement of Mitochondrial Membrane Potential

**Mitochondrial membrane potential** was determined using JC-1 assay kit (Solarbio, China). After 24 h of LPS (1 μg/mL) exposure, the cell culture was discarded and was replaced with JC-1 staining solution (100 ml/well). Then, they were incubated at 37°C for 30 min in darkness and washed with PBS, JC-1 fluorescence was detected and photographed using a LSM-800 with Airyscan. For red fluorescent of J-aggregates, the wavelength of excitation/emission were set as 525 and 590 nm, respectively, and for the fluoresce green of J-monomers, the excitation/emission were 490/530 nm, respectively.

### Statistical Analysis

The results are expressed as the mean ± standard deviation. The significant differences between the two groups were analyzed using unpaired *t*-tests. Data were analyzed by one-way ANOVA followed by Dunnett's test when the data involved three or more groups using GraphPad Prism 8.0.2.

## Results and Discussion

### Nutritional Value of *R. officinale* and *R. tanguticum* Stalks

#### Proximate Compositions

The proximate compositions of SRO and SRT are presented in Table 1. The most abundant components were carbohydrates with contents of 66.65 ± 5.23% for SRO and 59.39 ± 6.36% for SRT followed by ash. However, there were no significant differences between these two groups (*P* > 0.05). The stalks of the two species contained abundant fiber, exhibiting contents of 11.71 ± 1.66% for SRO and 13.17 ± 2.05% for SRT. From *R. ribes* and *R. rhabarbarum*, the natural active ingredients and dietary fiber also were found (7, 8). In addition, the protein contents were 6.06 ± 1.12% for SRO and 9.51 ± 0.98% for SRT, with a significant difference being detected (*p* < 0.05). The lipid contents of SRO and SRT were notably low. Due to the high level of carbohydrates, fiber and proteins, we thought that SRO and SRT could be widely utilized as feed additives to advance the digestion and provide proteins. However, the relationship between the proximate compositions and harvest seasons of SRO and SRT should be investigated further.

**Table 1:** The nutritional values of *R. officinale and R. tanguticum* stalks.

**Proximate compositions (%)**			**Minerals element (mg/100 g)**			**Amino acids (mg/100 g)**			**Fatty acids (mg/100 g)**		
**Name**	**SRO**	**SRT**	**Name**	**SRO**	**SRT**	**Name**	**SRO**	**SRT**	**Name**	**SRO**	**SRT**
Lipids	0.51 ± 0.03^a^	0.40 ± 0.03^b^	Calcium	3,280.1 ± 15.29^a^	4,280.3 ± 39.56^b^	Gly	281.23 ± 14.25^a^	480.32 ± 24.58^b^	C10	11.30 ± 2.11	–
Proteins	6.06 ± 1.12^a^	9.51 ± 0.98^b^	Sodium	47.60 ± 1.23^a^	67.45 ± 3.25^b^	Ala	273.21 ± 16.57^a^	490.42 ± 29.10^b^	C11	–	–
Moisture	7.31 ± 0.87^a^	5.90 ± 1.01^b^	Potassium	661.26 ± 10.11^a^	1,094.27 ± 9.56^b^	Val*	340.07 ± 21.45^a^	601.35 ± 19.89^b^	C12	77.76 ± 19.21^a^	18.73 ± 3.25^b^
Fiber	11.71 ± 1.66^a^	13.17 ± 2.05^a^	Iron	1.75 ± 0.09^a^	2.96 ± 0.25^b^	Leu*	401.33 ± 12.16^a^	800.83 ± 65.34^b^	C13	13.01 ± 1.12^a^	76.74 ± 13.12^b^
Ash	15.07 ± 2.17^a^	17.53 ± 2.12^a^	Phosphorus	230.05 ± 34.89^a^	240.67 ± 21.89^a^	Ile*	200.89 ± 12.14^a^	422.51 ± 9.54^b^	C14	19.32 ± 1.18^a^	19.26 ± 2.35^a^
Carbohydrates	66.65 ± 5.23^a^	59.39 ± 6.36^a^	Copper	0.17 ± 0.02^a^	0.16 ± 0.08^a^	Pro	790.09 ± 17.56^a^	1,050.12 ± 54.23^b^	C14:1	9.27 ± 1.12	–
			Manganese	10.32 ± 2.14^a^	5.39 ± 1.17^b^	Ser	311.26 ± 12.43^a^	503.25 ± 8.99^b^	C15	24.43 ± 3.21^a^	20.08 ± 4.51^a^
			Magnesium	43.60 ± 2.68^a^	61.62 ± 3.63^b^	Cys	70.01 ± 9.87^a^	210.90 ± 10.76^b^	C15:1	10.61 ± 1.23^a^	9.41 ± 2.12^a^
			Selenium	–	–	Met*	–	30.00 ± 3.00	C16	860.16 ± 56.81^a^	958.06 ± 72.31^a^
			Zinc	0.84 ± 0.12^a^	0.84 ± 0.09^a^	Thr*	233.21 ± 23.19^a^	450.09 ± 16.98^b^	C16:1	20.77 ± 3.12^a^	18.90 ± 1.89^a^
			Total	4,275.5 ± 56.23^a^	5,747.8 ± 32.15^b^	Phe*	180.09 ± 8.67^a^	121.11 ± 3.21^b^	C17	16.56 ± 3.45^a^	11.25 ± 5.21^a^
						Tyr	460.98 ± 24.89^a^	592.65 ± 35.89^b^	C18	83.63 ± 25.41^a^	72.30 ± 31.23^b^
						Asp	498.78 ± 15.87^a^	1,080.02 ± 87.23^b^	C18:1	680.28 ± 11.23^a^	509.28 ± 7.89^b^
						Glu	1,213.16 ± 36.12^a^	1,630.08 ± 76.28^b^	C18:2	1,617.16 ± 23.45^a^	1,936.4 ± 41.21^b^
						Lys*	330.09 ± 19.21^a^	581.31 ± 24.34^b^	C18:3	524.63 ± 67.23^a^	592.97 ± 52.31^a^
						Arg	290.00 ± 10.21^a^	563.21 ± 18.91^b^	C20	–	13.29 ± 2.45
						His*	150.02 ± 7.89^a^	221.21 ± 14.42^b^	C20:1	15.12 ± 1.32^a^	14.64 ± 3.41^a^
						Total	6,024.42 ± 93.25^a^	9,829.38 ± 72.13^b^	C20:2	–	123.07 ± 34.23
						E-total	1,835.70 ± 91.23^a^	3,228.41 ± 64.21^b^	C20:3	46.85 ± 45.71^a^	57.46 ± 34.51^b^
									C21	52.38 ± 18.89^a^	57.01 ± 23.12^a^
									C22:6	55.68 ± 9.21^a^	134.79 ± 18.21^b^
									C23	28.10 ± 3.12	–
									C24	82.20 ± 34.12^a^	109.93 ± 29.11^a^
									Total	4,249.22 ± 62.23^a^	4,753.57 ± 82.12^a^
									SFA	1,268.85 ± 30.11^a^	1,356.65 ± 22.31^a^
									MUFA	736.05 ± 28.35^a^	552.23 ± 36.78^a^
									PUFA	2,244.32 ± 40.10^a^	2,844.69 ± 24.16^b^

*SFA, saturated fatty acids; MUFA, monounsaturated fatty acids; PUFA, polyunsaturated fatty acids; E-total, The total contents of eight essential proteins and one protein (His) for children. Different letters denote of data significant difference at p <0.05 between the two group and those followed by same letters denote no significant difference*.

#### Mineral Compositions

As shown in Table 1, SRO and SRT contain abundant minerals with values of 4,275.5 mg/100 g and 5,747.8 mg/100 g samples, respectively. Among these minerals, the contents of Ca were the highest, exhibiting a concentration of 3,280.1 mg/100 g for SRO and 4,280.3 mg/100 g for SRT, which were higher than the recommended daily intakes of 1,000–1,300 mg/day (25). From New Zealand Nutrition databases, we observed that the standard unit in a cup size for rhubarb is 265 g (26). According to this report, the total content of Ca in raw rhubarb was 8.69 g/serving, which was higher than that in trim milk (1.67 g/serving) and standard milk (1.65 g/serving). From the results of this work, these two species could be employed as good food supplements and feed additives to provide Ca for humans and animals to improve bone health and other metabolic processes and to reduce the risk of osteoporosis. Next, the content of K was investigated, and the content in SRT (1,094.27 mg/100 g) was observed to be greater than that in SRO (661.26 mg/100 g) (*p* < 0.05). The level of minerals in plant can be attributed to the different habitat, plant lifespan, and soil condition. In the karst region of south-western China, the levels of Ca in senesced leaves of different plant functional types were higher than that of K (27), and the contents of Ca in *Ipomoea aquatica, Achyranthes aspera, Aasystasia ganjetica, Enhydra fluctuans, Oldenlandia corymbosa*, and *Amaranthus viridis* also were higher than that of K (28). Further studies should be carried out to investigate the mineral profile, pH, EC, and texture profile of soil in which the samples were cultivated, and to understand the possible reason why the content of Ca was higher than that of K. We also observed the lowest ratio of Na/K with values of 0.072 and 0.062, respectively, and the lower ratio of these two minerals may benefit cardiovascular health. SRO and SRT were subsequently detected with values of 230.05 and 240.67 mg/100 g for P and 43.60 and 61.62 mg/100 g for Mg, respectively, which are important for enabling antioxidant enzymes *in vivo* to protect the body from diseases. However, the contents of Zn and Cu were the lowest, and Se was not detected. Mineral elements play important roles in improving such body functions as bone and tooth performance, immune balance, and cardiovascular function.

#### Amino Acids Compositions

Amino acids are important for the formation of proteins, and their profile determines protein quality (29). In this work, we observed that the two species contained high levels of important amino acids, and fifteen and sixteen amino acids were detected from SRO and SRT, respectively. The total contents were 6,024.42 mg/100 g for SRO and 9,829.38 mg/100 g for SRT, respectively. These two species provided abundant nutrient substances to maintain the balance of the diet. Essential amino acids, which were only absorbed and obtained from the diet, are necessary for human health and cannot be synthesized by the body (30), and SRT has a higher content of 3,228.41 mg/100 g than SRO (1,835.70; *P* < 0.05). Glutamic acid was the most abundant amino acid, exhibiting contents of 1,213.16 mg/100 g for SRO and 1,630.08 mg/100 g for SRT. This result provide a clue that the stalks of two *Rheum* species could be applied in recipes where umamai flavor is desired. The level of glutamic acid is also highest among other amino acids in *Portulaca oleracea, Anoplophora chinensis*, and *Tenebrio molitor* (17, 18). The most abundant amino acids were leucine (401.33 vs. 800.83 mg/100 g, *p* < 0.05), valine (340.07 vs. 601.35 mg/100 g, *p* < 0.05), and lysine (330.09 vs 581.31 mg/100 g, *p* < 0.05) for the two species. In SRO, six essential amino acids were detected, and for SRT, seven amino acids were identified, including methionine. In addition, we also observed that the two species contained the most abundant hydrophobic amino acids, which have bioactive functions for reducing the risk of disease (31). These results indicated that SRO and SRT may represent useful sources of amino acids and food supplementation to protect the body by enhancing immune function.

#### Fatty Acids Compositions

As shown in Table 1, SRO and SRT were rich in fatty acids, exhibiting contents of 4,249.22 mg/100 g and 4,753.57 mg/100 g, respectively. In those studies, the contents of monounsaturated fatty acids (MUFAs) and polyunsaturated fatty acids (PUFAs) were determined to be 736.05 mg/100 g and 2,244.32 mg/100 g for SRO and 552.23 mg/100 g and 2,844.69 mg/100 g for SRT, which played important roles in maintaining the high quality of the human diet and food (17, 25) and could be employed to decrease the risk of heart disease in humans (32). Low-dose polyunsaturated fatty acids and omega-3 fatty acids supplementation attenuates inflammatory markers (33, 34). Humans cannot synthesize omega-3 and omega-6 fatty acids, which are only obtained from food (32, 35). Currently, the Western diet contains abundant omega-6 and a low content of omega-3 fatty acids, which leads to a high omega-6/omega-3 ratio (10–20:1) and induces the progression of some chronic autoimmune and degenerative diseases (36). Hence, low omega-6/omega-3 ratios, i.e., 1:1–4:1, are recommended and would be beneficial to human health for medicinal uses (37). In our work, the low and proper ratios of omega-6/omega-3 were also found with values of 2.82:1 and 2.98:1, and the essential fatty acids α-linolenic (18:3) and linoleic acids (18:2), and the total contents of fatty acids were 2,141.79 mg/100 g for SRO and 2,529.37 mg/100 g for SRT. This result suggested that the stalks of the two species may have cardioprotective, antiinflammatory, and antioxidant activities and may also reduce the risks of some cancers (38). The two fatty acids eicosapentaenoic acid and docosahexaenoic acid, two precursor of arachidonic acids, presented the cardiovascular protective effect and also could reduce the risks of some cancers (38). C18:1 could prevent insulin resistance and may have an antiinflammatory effect (39); it was also identified from SRO and SRT. This result indicated that SRO and SRT contained essential fatty acids and could be employed to prevent chronic cardiovascular diseases and regulate the immunity.

### UHPLC-QQQ-MS-Based Metabolomics

**Figure 1:**
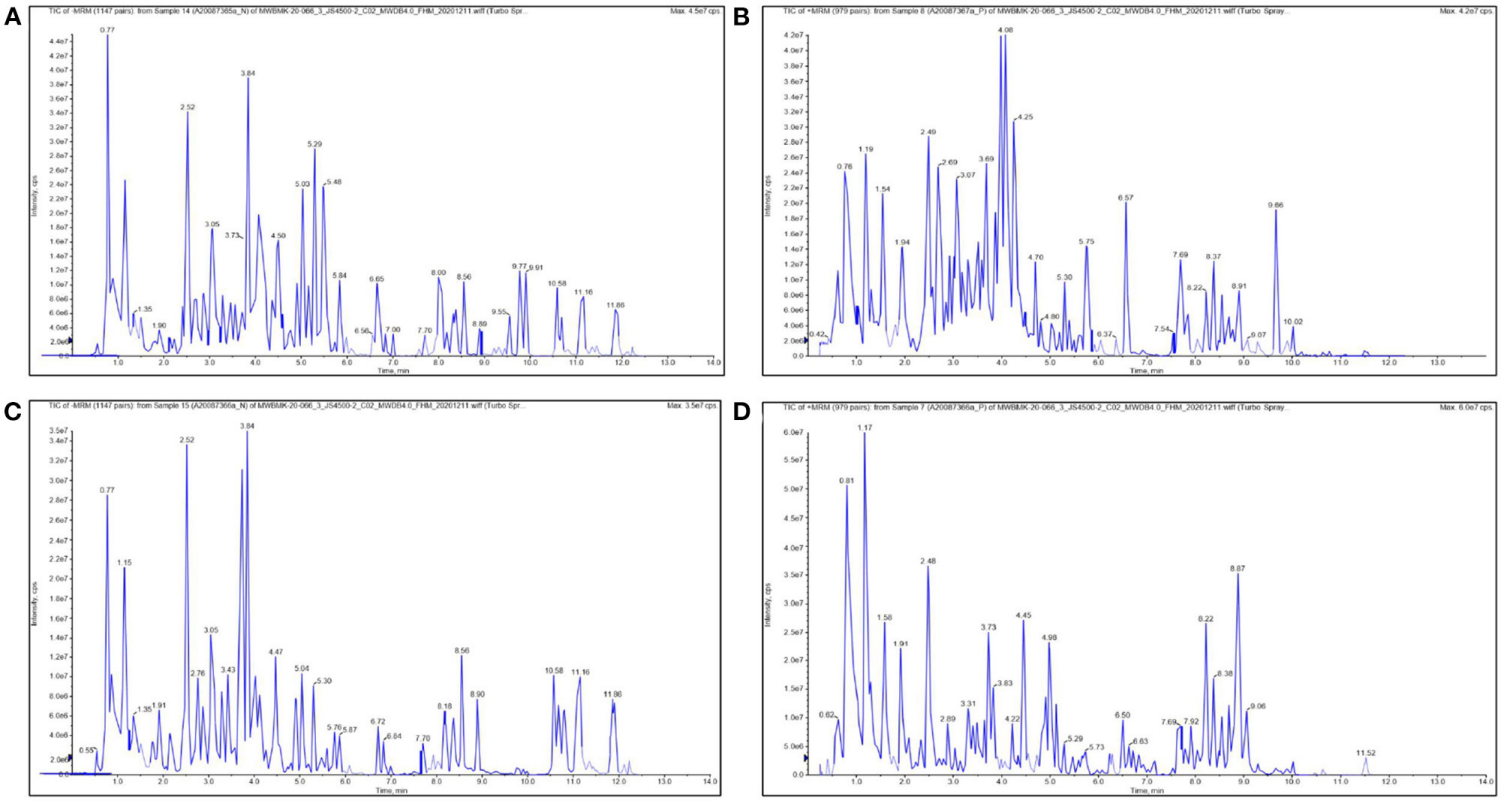
The chromatographic pictures of UHPLC-QQQ-MS-based metabolomics. [**(A)** negative ion for SRO; **(B)** positive ion for SRO; **(C)** negative ion for SRT; **(D)** positive ion for SRT].

**Figure 2:**
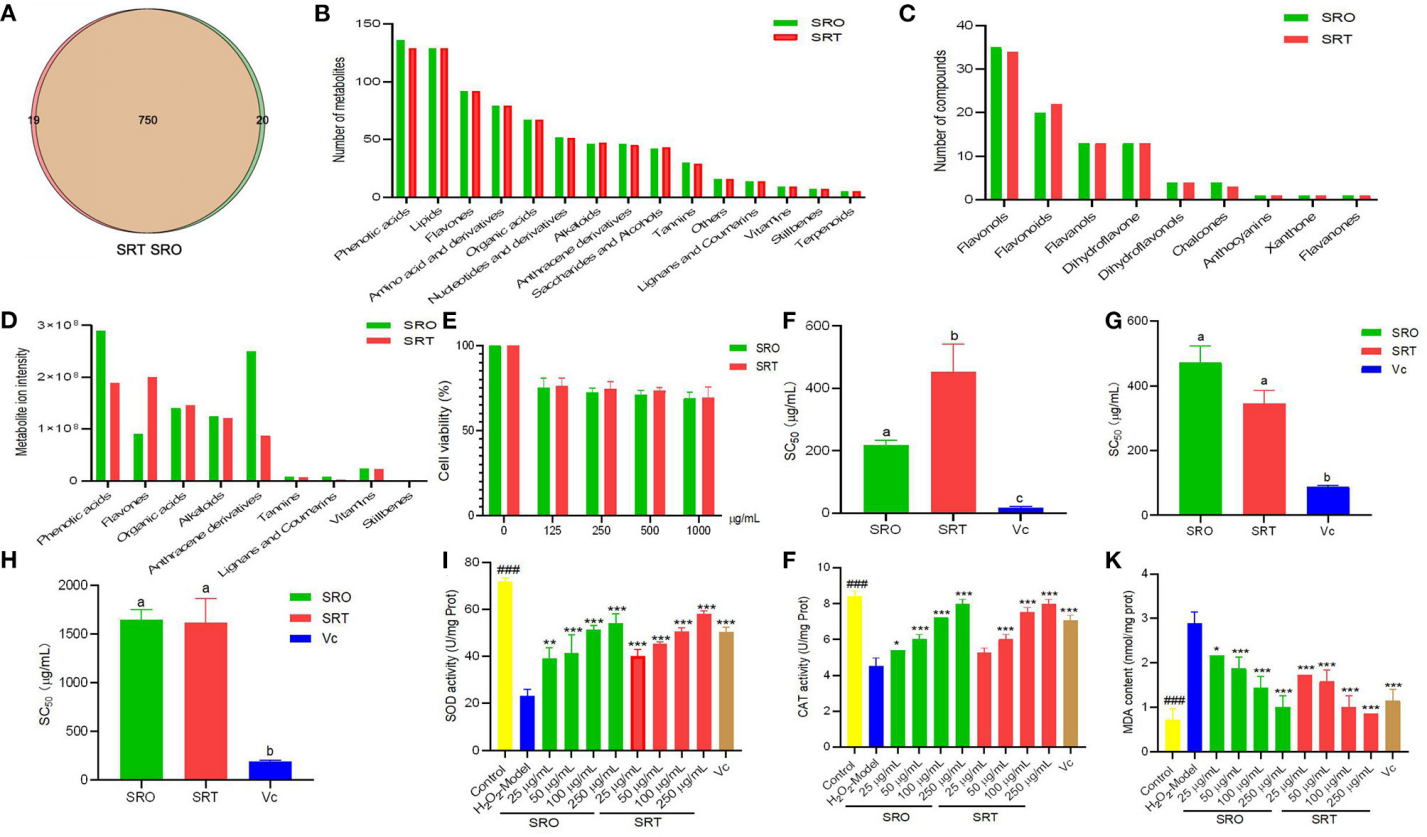
The number of compounds **(A)**, metabolites **(B)**, and flavones **(C)** in SRO and SRT, and the metabolite ion intensity of secondary in SRO and SRT **(D)**; the cell viability **(E)**; SC_50_ values for DPPH radical scavenging activity **(F)**, hydroxyl radical scavenging activity **(G)**, and superoxide radical scavenging activity **(H)**; the SOD activity **(I)**, CAT activity **(J)**, and MDA content **(K)** in RAW264.7 cells induced by H_2_O_2_ [For **(F–H)**, the different letter represents the significant difference, *p* < 0.05; for **(I–K)**, ^###^represent the significant difference between control group and model group, *p* < 0.001; *p < 0.05 represent the significant difference between model group and drug-treated group; **p < 0.01; *** for *p* < 0.001].

Consumption of traditional vegetables or plants can assist in the prevention of chronic disease development, as they contain various bioactive compounds that exhibit multiple health benefits (40). Using LC-MS-based metabolomics, the chemical compositions were analyzed (Figure 1), and 770 and 769 compounds were identified from SRO and SRT, respectively (Figure 2A). The chromatographic pictures are presented in Figure 1, and the data on tentative chemical compounds and molecular formula of compounds are described in Supplementary Table 1.

Among of all compounds, phenolic acids and lipids had the most compounds for SRO (136 and 129, respectively), and for SRT (129 and 129), and next was flavones (92 and 92), which includes a variety of chemical structure, flavonols, flavonoids, flavanols, dihydroflavone, dihydroflavonols, chalcones, anthocyanins, xanthone, and flavanones. Amino acid and derivatives (79 and 79), organic acids (67 and 67), nucleotides and derivatives (52 and 51), alkaloids (46 and 47), anthracene derivatives (46 and 45), saccharides and alcohols (42 and 43), and tannnins (30 and 29) from SRO and SRT, respectively, also were found (Figures 2B,C). In addition, they also contained some amount of vitamins. Hence, we thought that SRO and SRT could be used as vegetables due to the high nutritional values, such as amino acids, nucleotides, saccharides, fatty acids, and vitamins.

Subsequently, the relative ion intensity of secondary metabolites from SRO and SRT were analyzed and compared. Although the numbers of each kinds of compounds from SRO and SRT were similar, the relative intensity was different. Results showed that the highest content of SRO was phenolic acids, next were anthracene derivatives, organic acids, alkaloids and flavones; however, the most content of SRT is flavones, and others were phenolic acids, organic acids, alkaloids, and anthracene derivatives (Figure 2D). Table 2 presented that except the common compounds citric acid, isocitric acid, and caffeic acid, the main compounds are different between SRO and SRT. The different contents of SRO and SRT affect their flavor and taste, SRO is more bitter than SRT. The different components and contents would cause the different pharmacolog ical activities of SRO and SRT. Considering the high levels of flavones and phenolic acids, we thought SRT may be more suitable to be food additive than SRO.

**Table 2:** Top ten compounds identified from SRO and SRT.

**No**.	**Ion mode**	**Molecular Weight (Da)**	**Formula**	**Ionization model**	**Compounds**	**Class**
**SRO**						
1	Negative	432.106	C_21_H_20_O_10_	[M-H]^−^	Emodin-6-O-glucoside	Anthracene derivatives
2	Negative	192.027	C_6_H_8_O_7_	[M-H]^−^	Citric acid	Organic acids
3	Positive	121.089	C_8_H_11_N	[M+H]^+^	Phenethylamine	Alkaloids
4	Negative	408.142	C_20_H_24_O_9_	[M-H]^−^	Torachrysone-8-O-glucoside	Phenolic acids
5	Negative	510.131	C_30_H_22_O_8_	[M-H]^−^	Palmidin A	Anthracene derivatives
6	Negative	192.027	C_6_H_8_O_7_	[M-H]^−^	Isocitric Acid	Phenolic acids
7	Negative	246.089	C_14_H_14_O_4_	[M-H]^−^	Torachrysone	Phenolic acids
8	Negative	154.027	C_7_H_6_O_4_	[M-H]^−^	Gentisic acid	Phenolic acids
9	Negative	180.042	C_9_H_8_O_4_	[M-H]^−^	Caffeic acid	Phenolic acids
10	Positive	141.09	C_6_H_11_N_3_O	[M+H]^+^	Histidinol	Phenolic acids
**SRT**						
1	Negative	432.106	C_21_H_20_O_10_	[M-H]^−^	Isovitexin	Flavonoids
2	Negative	432.106	C_21_H_20_O_10_	[M-H]^−^	Vitexin	Flavonoids
3	Positive	313.131	C_18_H_19_NO_4_	[M+H]^+^	N-Feruloyltyramine	Alkaloids
4	Negative	192.027	C_6_H_8_O_7_	[M-H]^−^	Citric Acid	Organic acids
5	Positive	187.063	C_11_H_9_NO_2_	[M+H]^+^	3-Indoleacrylic acid	Alkaloids
6	Negative	432.106	C_21_H_20_O_10_	[M-H]^−^	Emodin-6-O-glucoside	Anthracene derivatives
7	Negative	304.058	C_15_H_12_O_7_	[M-H]^−^	Dihydroquercetin	Flavonoids
8	Negative	180.042	C_9_H_8_O_4_	[M-H]^−^	Caffeic acid	Phenolic acids
9	Negative	192.027	C_6_H_8_O_7_	[M-H]^−^	Isocitric acid	Organic acids
10	Negative	154.027	C_7_H_6_O_4_	[M-H]^−^	Protocatechuic acid	Phenolic acids

### Toxicity

To ensure the safety of the two species, we evaluated the acute toxicity in mice. After oral administration of SRO and SRT (5,000 mg/kg) to mice, no mice died or exhibited any acute behavior, and the LD_50_ was determined to be more than 5,000 mg/kg. From Figure 2E, we can see that the two stalks presented the weak cytotoxicity against RAW 264.7 cells. Even at 1,000 μg/mL, the cell viability of two plants were more than 75 %. This result suggested that at the concentration employed in this work, the two species were safe.

### Antioxidant Activity

#### *In vitro* Antioxidant Activity

The antioxidant activities of the two species were evaluated and compared using three different assays. From Figure 2F, we can see that SRO presented stronger radical scavenging activity than SRT in the DPPH assay (*p* < 0.05), which was employed to investigate the reducing power of agents based on an electron transfer reaction. The EC_50_ values were 205.13 and 497.03 μg/mL, respectively, and the positive control Vc was 19.33 μg/mL. However, the superoxide radical scavenging activity of SRT was better than that of SRO (*p* < 0.05; Figure 2G). This result indicated that the two species could alleviate the cellular damage induced by the superoxide radical and could contribute to aging and some degenerative diseases.

Hydroxyl radicals can cause severe cell death or damage by crossing cell membranes and subsequently reacting with biomacromolecules (24). However, the hydroxyl radical activity of the two species was weak, and the EC_50_ values were more than 1,000 μg/mL (Figure 2H). These results suggested that the two species have antioxidant activity *in vivo*, especially for radical scavenging activity in the DPPH assay and superoxide radical scavenging activity.

#### Cellular Antioxidant Activity

Macrophages stimulated by H_2_O_2_ would cause the enrichment of superoxide radicals and other radicals. SOD and CAT as enzymatic antioxidant defenses provide first-line cellular protection contributing to prevent cellular damage and maintain a balance between free radical production and oxidative stress after being stimulated by oxidative stress (41, 42). Most plant antioxidants are investigated for the effects by evaluating directly the enzymatic activity of endogenous antioxidants (43).

A ubiquitous cellular oxidant, H_2_O_2_, is mostly produced by the peroxisome. Figure 2I showed that after the treatment of H_2_O_2_, the activity of SOD was significant inhibited (*P* < 0.001); however, SRO and SRT dramatically activated SOD activity. At the concentrations of 50, 100, and 250 μg/mL of the samples, the SOD activities were 41.47, 51.31, and 54.24 U/mg Prot (protein) for SRO, 45.45, 50.47, and 58.01 U/mg Prot for SRT (all *P*<*0.001*); and the SOD activity was 50.37 U/mg Prot for the positive control (10 μg/mL). In addition, after treatment with SRO and SRT, CAT activity was increased in RAW264.6 cells induced by LPS in a dose-dependent manner (Figure 2J). The activation in SOD and CAT activities contribute to scavenge free radicals and other radicals from macrophages induced by H_2_O_2_. As a marker of protein and lipid oxidation, the cellular MDA levels would be released when they respond to oxidant stress (44). From Figure 2K, we can see that the two stalks could reduce the increased production of MDA in cells stimulated by LPS in a dose-dependent manner, respectively. Especially for SRT, the MDA contents were significant lower than model group (*p* < 0.001). These results indicated that SRO and SRT have the good cellular antioxidant activity.

### Antiinflammatory Activity

**Figure 3:**
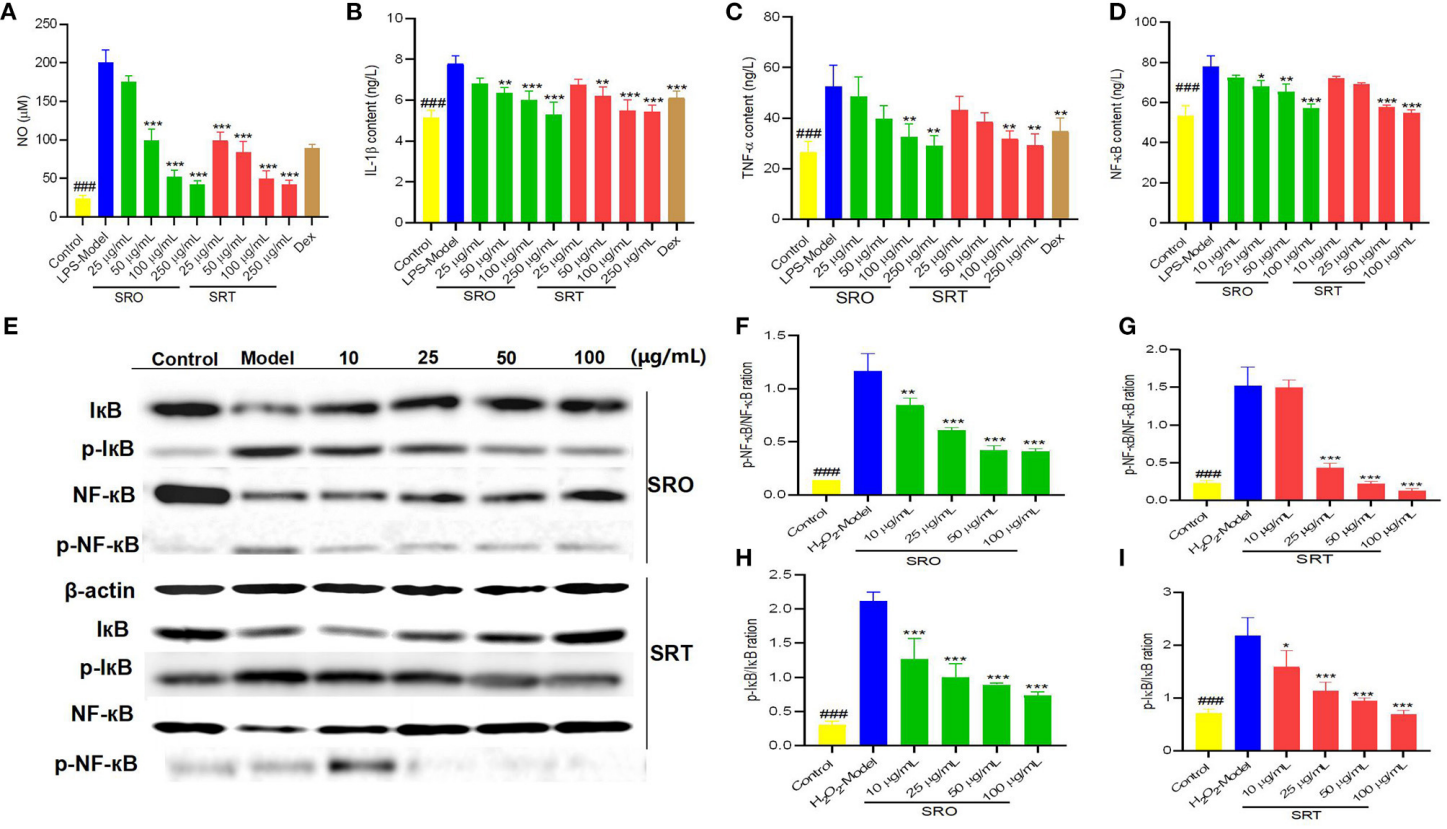
The NO content **(A)**, IL-1β content **(B)**, TNF-α content **(C)**, and NF-κB content **(D)** in RAW264.7 cells induced by LPS; the western blot assay for determining the expression of IκB and NF-κB **(E)**; the p-NF-κB/NF-κB ration for SRO **(F)** and SRT **(G)**; the p-IκB/IκB ration for SRO **(H)** and SRT **(I)** (^###^represent the significant difference between control group and model group, *p* < 0.001; *p < 0.05 represent the significant difference between model group and drug-treated group; **p < 0.01; ***p < 0.001).

When stimulated by LPS and other foreign substances, macrophages play an important role in inflammatory processes and release various cytokines. As one of a well-known inflammatory cytokine, NO is released by activated macrophages and has a cytotoxic effect through the formation of peroxynitrite with superoxide anion and inflammatory response (45). Figure 3A demonstrated that after LPS treatment, NO was stimulated and released (201.00 μM); however, two plants reduced the content of NO in RAW 264.7 cells. At the concentrations of 25, 50, 100, and 250 μg/mL, the levels of NO were 175.33, 99.67, 52.00, and 42.67 μM for SRO, and 99.67, 84.33, 50, and 42.33 μM for SRT, respectively. In addition, SRO and SRT presented the similar inhibitory effects against the IL-1β production in a dose-dependent manner, especially for 50, 100, and 250 μg/mL (*p* < 0.01), which participates in the immune response and tissue repair in response to inflammatory diseases (Figure 3B). TNF-α plays an important role in inflammation formation. Compared with the model group (52.5 ng/L), the levels of TNF-α were markedly decreased when coincubated with SRO and SRT. Especially for 100 and 250 μg/mL (all *p* < 0.01; Figure 3C). The results showed that the two stalks could decrease the production of proinflammatory cytokines stimulated by LPS in a dose-dependent manner, and SRT presented better antiinflammatory activity than SRO.

### Measurement of NF-κB p65 Content

NF-κB is a nearly ubiquitous pathway responsible for mediating DNA transcription that is involved in apoptosis, proliferation, and metastasis (46). When a cell receives any of a multitude of extracellular signals, NF-κB rapidly enters the nucleus and activates gene expression. Figure 3D demonstrated that compared with model group (77.83 ng/L), SRO and SRT could decrease the levels of NF-κB p65 in RAW264.7 cells induced by LPS in a dose-dependent manner. Especially for high concentration (100 μg/mL), the contents were 57.27 ng/L for SRO and 54.90 ng/L for SRT (*p* < 0.001), respectively. These results indicated that the two stalks presented the antiinflammatory activity by decreasing the level of NF-κB and then inhibiting the activation of NF-κB pathway in RAW264.7 cells induced by LPS.

### Western Blot Analysis

The western blot test was conducted to investigate the expression of IκB and NF-κB in NF-κB signaling pathway for explaining the possible antiinflammatory mechanism. Compared with the model group, SRO and SRT reduced the expression ratio of phosphorylation of IκB/IκB and the phosphorylation of NF-κB/NF-κB of RAW264.7cells induced by LPS in a dose-dependent manner, especially at the concentrations of 25, 50, and 100 μg/mL (*p* < 0.001; Figures 3E–I). Cytokines mediate the rapid activation of NFκB through activation of the IKK complex, lead to subsequent phosphorylation and degradation of the inhibitory IκB proteins, then stimulate activation of NFκB, and transcription of target genes (47). A key step for controlling NF-κB activity and its phosphorylation is the regulation of the IkB-NF-κB interaction. Hence, we thought that SRO and SRT presented the antiinflammatory activity by inhibiting NF-κB signaling pathway and decreasing the cytokines production.

### Measurement of the Cellular ROS Production

**Figure 4:**
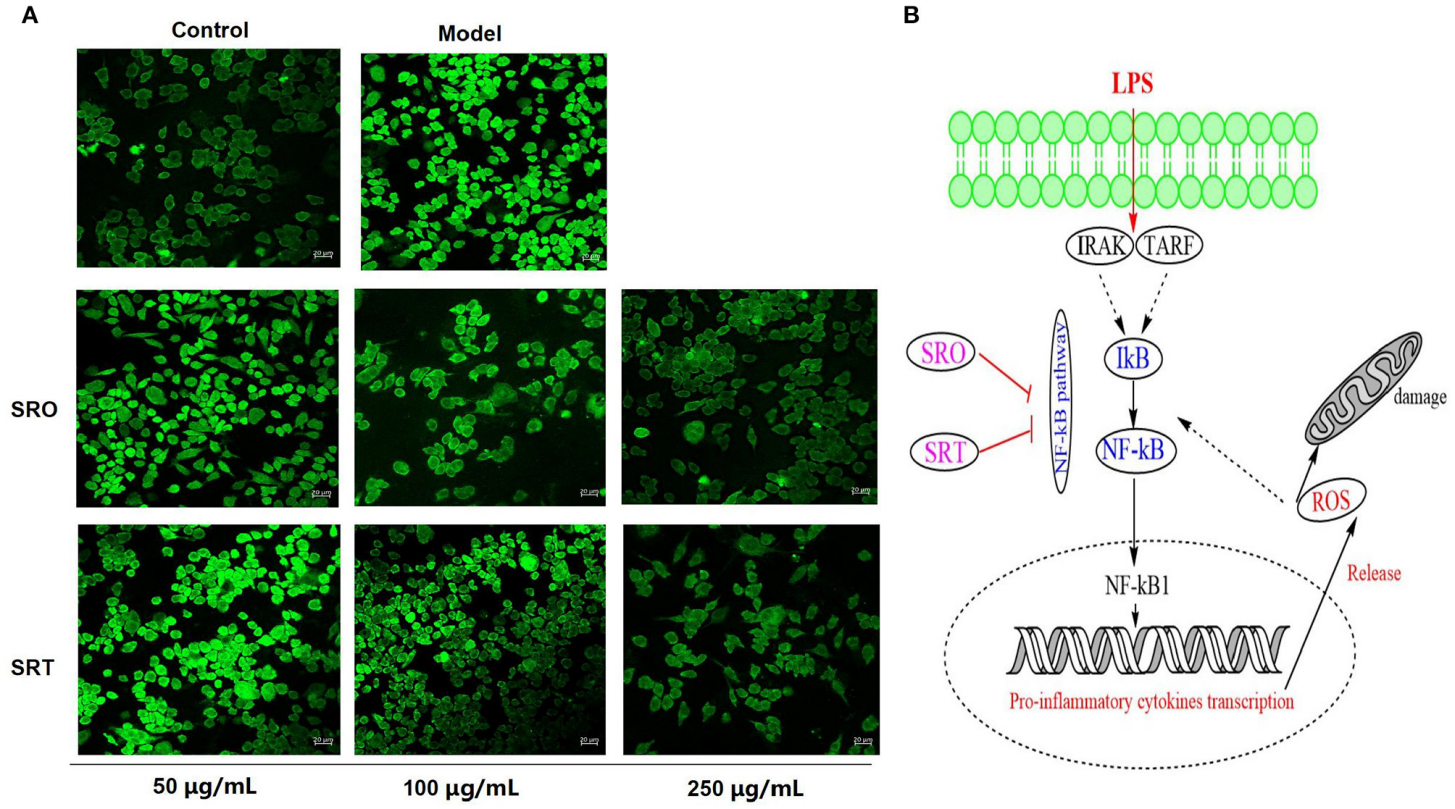
The fluorescence for measuring the cellular ROS production **(A)** and the NF-κB signaling pathway **(B)** of RAW264.7 cells treated by SRO and SRT.

The oxidant and inflammatory response to exogenous substance would increase cellular ROS production and accumulation. From Figure 4A, we can see that compared with the control group, the intensity of green fluorescence stained by DCFH-DA in model group was significantly enhanced, and LPS could generate ROS production of RAW264.7 cells. However, after the treatment of SRO and SRT, the green fluorescence became vague, respectively. The intensity of two groups were decreased in a dose-dependent manner, especially for 100 and 250 μg/mL. These results illustrated that the two stalks could inhibit the ROS production and accumulation of RAW264.7 cells induced by LPS (Figure 4B).

### Measurement of the Cellular MMP

**Figure 5:**
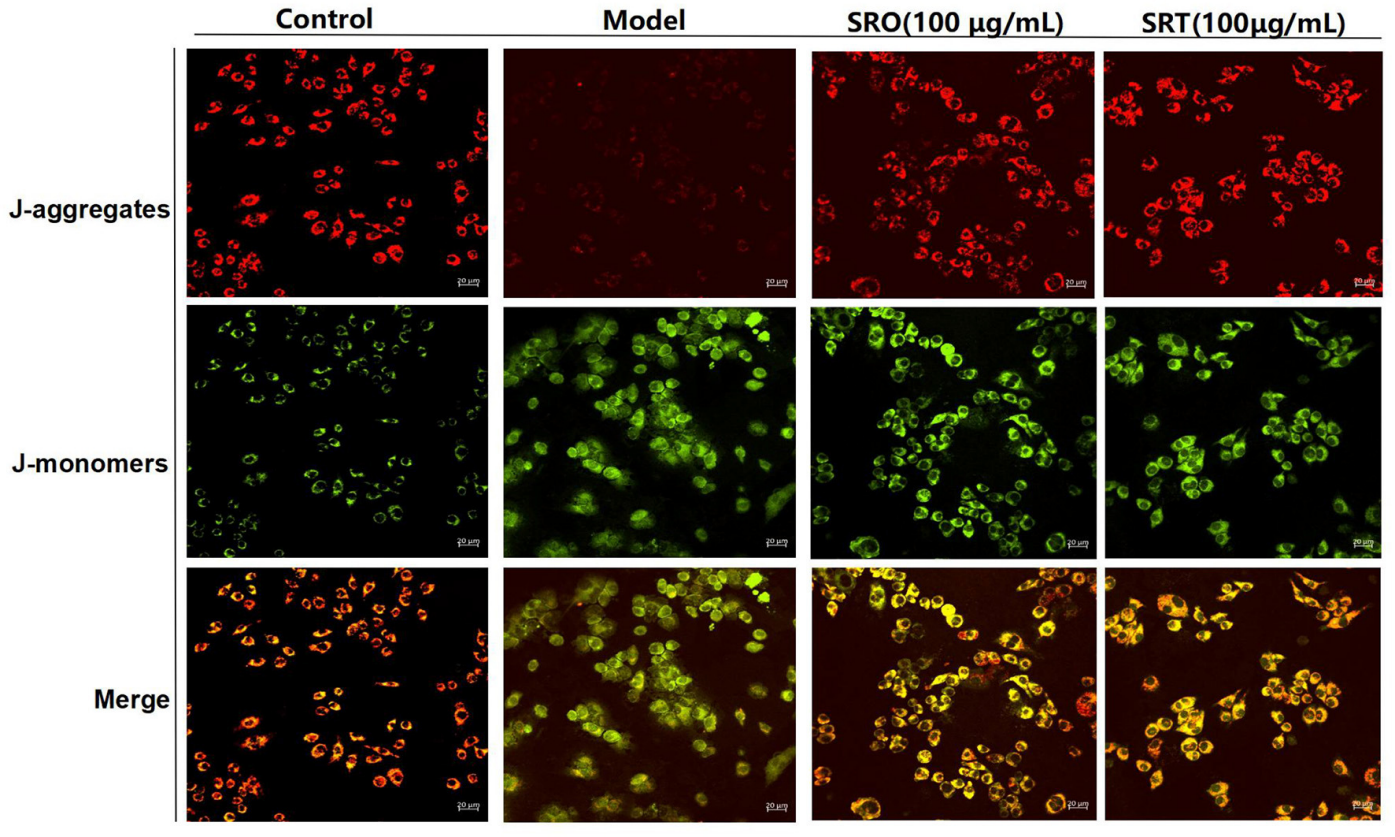
The fluorescence for indicating the cellular MMP of RAW264.7 cells treated by SRO and SRT.

The generation and accumulation of ROS cause the reduction of MMP and the increase of cell membrane permeability, also resulting in cell death. In this test, JC-1 kits were used to evaluate the effect against MMP. JC-1 staining showed an aggregated pattern of energized mitochondria (red fluorescence) and a monomeric pattern for depolarized mitochondria (green fluorescence). As shown in Figure 5, compared with control group, RAW264.7 cells induced by LPS presented the remarkably lower red fluorescence and higher green fluorescence, resulting in the loss of MMP. However, after the treatment of SRO and SRT at 100 μg/mL, the intensity of red and green fluorescence were reversed, respectively, and RAW264.7 cells showed higher red fluorescence and lower green fluorescence. These results indicated that the two plants have the significant inhibitory effect on MMP depletion induced by LPS.

### The Structure–Function Relationship of Food Bioactives and Ingredients

The applications and biological activities of foods and plants and their ingredients depend on the structure of those constituents, and the investigation of structure–function relationship of food bioactives and ingredients has attracted many people's attention. In this work, the nutritional values of the two stalks were found; however, SRT has higher properties than SRO, such as fiber, proteins, Ca, amino acids, fatty acids, and other key indexes. In addition, the different secondary compositions of the two stalks resulted in different activities. Similar to most compositions of SRO and SRT, phenolic acids are a key class of dietary polyphenols and natural antioxidants with healthy benefits, and they are precursors of other significant bioactive molecules regularly used for therapeutics, cosmetics, and food industries (47, 48). Hence, the two stalks could be used as healthy foods due to the high nutritional values and phenolic acids. In addition, flavones have a significant positive effect on diseases related to oxidative stress and inflammatory by regulating multiple cell signaling pathways and alterations in multiple cellular signaling pathways, such as NF-κB signaling pathway (49), and anthracene derivatives have the cytotoxicity and antiviral properties (50). Hence, SRT has better antioxidant and antiinflammatory activities than SRO.

Further studies indicated that the two stalks alleviated the oxidant stress and inflammatory response by inhibiting NF-κB signaling pathway in a dose-dependent manner. The overexpression of NF-κB and IκB were blocked by SRO and SRT, the damage of MMP and the accumulation of ROS were recovered, and then the production of cytokines was inhibited.

## Conclusion

*R. officinale* and *R. tanguticum* stalks have good nutritional value. The two species are rich in minerals, essential amino acids, and essential fatty acids. They possess abundant fiber and proteins and are used to regulate the immunity and enhance the immune function of body. Since the secondary and its levels between SRO and SRT were different, the bioactivies of the two stalks were various. SRT contains more flavones and phenolic acids with the better antiinflammatory activities than SRO. The further studies showed that the two stalks alleviated inflammatory response by inhibiting NF-κB signaling pathway. The antioxidiant activity also was observed. In addition, the good safety also was observed. The two stalks could be regarded as a potential new source of nutritional ingredients for the food industry. Further research should be conducted to elucidate the relationship between harvest season and active ingredients, and the digestibility and safety of the stalks of these species should also be investigated in the future.

## Data Availability Statement

The original contributions presented in the study are included in the article/Supplementary Material, further inquiries can be directed to the corresponding author/s.

## Author Contributions

X-FS: conceptualization, methodology, supervision, and funding acquisition. XM and X-RY: data analysis and writing-original draft. L-XD, L-PZ, Z-HL, and BL: investigation. F-YY and XG: data curation. YW: soft. J-YZ: validation. All authors contributed to the article and approved the submitted version.

## Funding

This work was financed by the Central Public-interest Scientific Institution Basal Research Fund (Grant No. 1610032021005 and 1610322021012), Innovation Project of Chinese Academy of Agricultural Sciences (Grant No. CAAS-ASTIP-2015-LIHPS), Natural Science Fund of Gansu Provincial Science and Technology Project (20JR10RA023), and China Agriculture Research System (CARS-37).

## Conflict of Interest

The authors declare that the research was conducted in the absence of any commercial or financial relationships that could be construed as a potential conflict of interest.

## Publisher's Note

All claims expressed in this article are solely those of the authors and do not necessarily represent those of their affiliated organizations, or those of the publisher, the editors and the reviewers. Any product that may be evaluated in this article, or claim that may be made by its manufacturer, is not guaranteed or endorsed by the publisher.

## Supplementary Material

Supplementary Table 1 The data on tentative chemical compounds and molecular formula of all compounds of SRO and SRT.

## References

[1] Zheng J Tian W Yang C Shi W Cao P Long J. Identification of flavonoids in *Plumula nelumbinis* and evaluation of their antioxidant properties from different habitats. Ind Crop Prod. (2019) 127:36–45. 10.1016/j.indcrop.2018.08.020

[2] Committee for the Pharmacopoeia of PR China. Pharmacopoeia of PR China, Part I. Beijing: China Medical Science and Technology Press (2010).

[3] The United States Pharmacopeia Convention. The United States Pharmacopoeia, Dietary Supplements Chapter. Baltimore, MD: United Book Press Inc (2010).

[4] European Directorate for the Quality of Medicines & HealthCare. Council of Europe. European Pharmacopoeia. 4th ed. Strasbourg: European Directorate for the Quality of Medicines & HealthCare (2001).

[5] Shang XF Zhao ZM Li JC Yang GZ Liu YQ Dai LX. Insecticidal and antifungal activities of *Rheum palmatum* L. anthraquinones and structurally related compounds. Ind Crop Prod. (2019) 137:508–20. 10.1016/j.indcrop.2019.05.055

[6] Gecibesler IH Disli F Bayindir S Toprak M Tufekci AR Yaglioglu AS. The isolation of secondary metabolites from *Rheum ribes* L. and the synthesis of new semi-synthetic anthraquinones: Isolation, synthesis and biological activity. Food Chem. (2020) 342:128378. 10.1016/j.foodchem.2020.128378 33508903

[7] Kalisz S Oszmiański J Kolniak-Ostek J Grobelna A Kieliszek M Cendrowski A. Effect of a variety of polyphenols compounds and antioxidant properties of rhubarb (*Rheum rhabarbarum*). LWT Food Sci Tech. (2020) 118:108775. 10.1016/j.lwt.2019.108775

[8] Nguyen HH Savage GP. Oxalate bioaccessibility in raw and cooked rhubarb (*Rheum Rhabarbarum* L.) during *in vitro* digestion. J Food Comp Anal. (2020) 94:103648. 10.1016/j.jfca.2020.103648

[9] Son SM Moon KD Lee CY. Rhubarb juice as a natural antibrowning agent. J Food Sci. (2000) 65:1288–9. 10.1111/j.1365-2621.2000.tb10598.x

[10] Xie C Song J Suo F Li X Li Y Yu H. Natural resource monitoring of *Rheum tanguticum* by multilevel remote sensing. Evid Based Compl Alter Med. (2014) 2014:618902. 10.1155/2014/618902 25101134 PMC4101945

[11] Ding LW. Analysis on the production and sales in market of rhubarb. Mod Chin Med. (2011) 13:56. 10.13313/j.issn.1673-4890.2011.09.011

[12] Xie L Wang Y Luo G Zhou W Miao J Tang S. Identification of the multiple bioactive derivatives and their endogenous molecular targets that may mediate the laxative effect of rhubarb in rats. J Trad Chin Med Sci. (2020) 7:210–20. 10.1016/j.jtcms.2020.04.004

[13] Liu Y Li L Xiao YQ Yao JQ Li PY Yu DR. Global metabolite profiling and diagnostic ion filtering strategy by LC-QTOF MS for rapid identification of raw and processed pieces of *Rheum palmatum* L. Food Chem. (2016) 192:531–40. 10.1016/j.foodchem.2015.07.013 26304381

[14] AOAC. Official Methods of Analysis of AOAC International. 21st Ed. Rockville, MD: AOAC. (2019)

[15] National Standardization Administration State Administration for Market Regulation. National Standards of the People's Republic of China (GB/T 6435-2014), Determination of moisture in feedstuffs (ISO6469:1999, MOD). Beijing: National Standardization Administration, State Administration for Market Regulation (2015).

[16] National Standardization Administration State Administration for Market Regulation. National Standards of the People's Republic of China (GB/T 6438-2007/ISO 5984:2002), Animal feeding stuffs-Determination of crude ash (ISO 5984:2002, IDT). Beijing: National Standardization Administration, State Administration for Market Regulation (2007).

[17] Wu RA Ding Q Yin L Chi X Sun N He R. Comparison of the nutritional value of mysore thorn borer (*Anoplophora chinensis*) and mealworm larva (*Tenebrio molitor*): Amino acid, fatty acid, and element profiles. Food Chem. (2020) 323:126818. 10.1016/j.foodchem.2020.126818 32330649

[18] Nemzer B Al-Taher F Abshiru N. Phytochemical composition and nutritional value of different plant parts in two cultivated and wild purslane (*Portulaca oleracea* L.) genotypes. Food Chem. (2020) 320:126621. 10.1016/j.foodchem.2020.126621 32203838

[19] Nie H Chen H Li G Su K Song M Duan Z. Comparison of flavonoids and phenylpropanoids compounds in Chinese water chestnut processed with different methods. Food Chem. (2021) 335:127662. 10.1016/j.foodchem.2020.127662 32739819

[20] Chu C Du Y Yu X Shi J Yuan X Liu X. Dynamics of antioxidant activities, metabolites, phenolic acids, flavonoids, and phenolic biosynthetic genes in germinating Chinese wild rice (*Zizania latifolia*). Food Chem. (2020) 318:126483. 10.1016/j.foodchem.2020.126483 32126468

[21] Merly L Smith SL. Murine RAW 264.7 cell line as an immune target: are we missing something? Immunopharmacol Immunotoxicol. (2017) 39:55–8. 10.1080/08923973.2017.1282511 28152640

[22] Zhang H Guo Q Liang Z Wang M Wang B Sun-Waterhouse D. Anti-inflammatory and antioxidant effects of chaetoglobosin Vb in LPS-induced RAW264.7 cells: achieved via the MAPK and NF-κB signaling pathways. Food Chem Toxicol. (2021) 147:111915. 10.1016/j.fct.2020.111915 33285210

[23] Ji Z Mao J Chen S Mao J. Antioxidant and anti-inflammatory activity of peptides from foxtail millet (*Setaria italica*) prolamins in HaCaT cells and RAW264.7 murine macrophages. Food Biosci. (2020) 36:100636. 10.1016/j.fbio.2020.100636

[24] Dai L He J Miao X Guo X Shang X Wang W. Multiple biological activities of *Rhododendron przewalskii* Maxim. extracts and UPLC-ESI-Q-TOF/MS characterization of their phytochemical composition. Front Pharmacol. (2021) 12:599778. 10.3389/fphar.2021.599778 33732152 PMC7957927

[25] FAO WHO. Vitamin and Mineral Requirements in Human Nutrition. 2nd edn. Rome: World Health Organization and Food and Agriculture Organization of the United Nations (2004).

[26] The Concise NZ Food Composition Tables. (2019). Retrieved from: https://www.foodcomposition.co.nz/foodfiles/concise-tables/

[27] Liu C Liu Y Guo K Wang S Yang Y. Concentrations and resorption patterns of 13 nutrients in different plant functional types in the karst region of south-western China. Ann Bot. (2014) 113:873–85. 10.1093/aob/mcu005 24573643 PMC3962245

[28] Datta S Sinha BK Bhattacharjee S Seal T. Nutritional composition, mineral content, antioxidant activity and quantitative estimation of water soluble vitamins and phenolics by RP-HPLC in some lesser used wild edible plants. Heliyon. (2019) 5:e01431. 10.1016/j.heliyon.2019.e01431 30976701 PMC6441826

[29] Van Huis A. Potential of insects as food and feed in assuring food security. Ann Rev Entomol. (2013) 58:563–83. 10.1146/annurev-ento-120811-153704 23020616

[30] Xiao F Guo F. Impacts of essential amino acids on energy balance. Mol Metab. (2021) 13:101393. 10.1016/j.molmet.2021.101393 34785395 PMC8829800

[31] Ding Q Zhang T Niu S Cao F Wu-Chen RA Luo L. Impact of ultrasound pretreatment on hydrolysate and digestion products of grape seed protein. Ultrasonics Sonochem. (2018) 42:704–13. 10.1016/j.ultsonch.2017.11.027 29429721

[32] Dorni C Sharma P Saikia G Longvah T. Fatty acid profile of edible oils and fats consumed in India. Food Chem. (2018) 238:9–15. 10.1016/j.foodchem.2017.05.072 28867107

[33] Tartibian B Maleki BH Abbasi A. Omega-3 fatty acids supplementation attenuates inflammatory markers after eccentric exercise in untrained men. Clin J Sport Med. (2011) 21:131–7. 10.1097/JSM.0b013e31820f8c2f 21358504

[34] Schubert R Kitz R Beermann C Rose MA Baer PC Zielen S. Influence of low-dose polyunsaturated fatty acids supplementation on the inflammatory response of healthy adults. Nutrition. (2007) 23:724–30. 10.1016/j.nut.2007.06.012 17664057

[35] Oboh A Kabeya N Carmona-Antoñanzas G Castro LFC Dick JR Tocher DR. Two alternative pathways for docosahexaenoic acid (DHA, 22:6n-3) biosynthesis are widespread among teleost fish. Sci Rep. (2017) 7:3889. 10.1038/s41598-017-04288-2 28634391 PMC5478668

[36] Sandre PC da Silva Chagas L de Velasco PC Galvani RG Dias Fraga KY Tavares do Carmo MDG. Chronic nutritional restriction of omega-3 fatty acids induces a pro-inflammatory profile during the development of the rat visual system. Brain Res Bull. (2021) 174:366–78. 10.1016/j.brainresbull.2021.07.001 34237395

[37] Simopoulos AP. The importance of the omega-6/omega-3 fatty acid ratio in cardiovascular disease and other chronic diseases. Exp Biol Med. (2008) 233:674–88. 10.3181/0711-MR-311 18408140

[38] D‘Eliseo D Velotti F. Omega-3 fatty acids and cancer cell cytotoxicity: implications for multi-targeted cancer therapy. J Clin Med. (2016) 5:15. 10.3390/jcm5020015 26821053 PMC4773771

[39] Palomer X Pizarro-delgado J Barroso E Vázquez-carrera M. Palmitic and oleic acid: the yin and yang of fatty acids in type 2 diabetes mellitus. Trends Endocrinol Metab. (2018) 29:178–90. 10.1016/j.tem.2017.11.009 29290500

[40] Sivakumar D Phan ADT Slabbert RM Sultanbawa Y Remize F. Phytochemical and nutritional quality changes during irrigation and postharvest processing of the underutilized vegetable african nightshade. Front Nutr. (2020) 7:576532. 10.3389/fnut.2020.576532 33304915 PMC7701055

[41] Sahlan M Mahira KF Pratami DK Rizal R Ansari MJ Al-Anazi KM. The cytotoxic and anti-inflammatory potential of Tetragonula sapiens Propolis from Sulawesi on Raw 264.7 cell lines. J King Saud Univ Sci. (2020) 33:101314. 10.1016/j.jksus.2020.101314

[42] Zhou H Yang J Xin T Li D Guo J Hu S. Exendin-4 protects adipose-derived mesenchymal stem cells from apoptosis induced by hydrogen peroxide through the PI3K/Akt–Sfrp2 pathways. Free Radical Bio Med. (2014) 77:363–75. 10.1016/j.freeradbiomed.2014.09.033 25452142

[43] Hu W Zhou J Shen T Wang X. Target-guided isolation of three main antioxidants from *Mahonia bealei* (Fort.) Carr. leaves using HSCCC. Molecules. (2019) 24:1907. 10.3390/molecules24101907 31108973 PMC6572348

[44] Kohen R Nyska A. Oxidation of biological systems: oxidative stress phenomena, antioxidants, redox reactions, and methods for their quantification. Toxicol Pathol. (2002) 30:620–50. 10.1080/01926230290166724 12512863

[45] Hilliard A Mendonca P Soliman KFA. Involvement of NF?B and MAPK signaling pathways in the preventive effects of *Ganoderma lucidum* on the inflammation of BV-2 microglial cells induced by LPS. J Neuroimmunol. (2020) 345:577269. 10.1016/j.jneuroim.2020.577269 32480240 PMC7382303

[46] Somade OT Ugbaja RN Alli AA Odubote OT Yusuf TS Busari BT. Diallyl disulfide, an organo-sulfur compound in garlic and onion attenuates trichloromethane-induced hepatic oxidative stress, activation of NFkB and apoptosis in rats. J Nutrit Intermed Metabol. (2018) 13:10–9. 10.1016/j.jnim.2018.07.005

[47] Kumar N Goel N. Phenolic acids: natural versatile molecules with promising therapeutic applications. Biotechnol Rep. (2019) 24:e00370. 10.1016/j.btre.2019.e00370 31516850 PMC6734135

[48] Rashmi HB Negi PS. Phenolic acids from vegetables: a review on processing stability and health benefits. Food Res Int. (2020) 136:109298. 10.1016/j.foodres.2020.109298 32846511

[49] Singh M Kaur M Silakari O. Flavones: an important scaffold for medicinal chemistry. Eur J Med Chem. (2014) 84:206–39. 10.1016/j.ejmech.2014.07.013 25019478

[50] Memeo MG Lapolla F Maga G Quadrelli P. Synthesis and antiviral activity of anthracene derivatives of isoxazolino-carbocyclic nucleoside analogues. Tetrahedron Lett. (2015) 56:1986–90. 10.1016/j.tetlet.2015.02.114

