# Supplementary figures and images for: High Value-Added Application of Two Renewable Sources as Healthy Food: The Nutritional Properties, Chemical Compositions, Antioxidant, and Antiinflammatory Activities of the Stalks of Rheum officinale Baill. and Rheum tanguticum Maxim. ex Regel
Source: Front Nutr. 2022 Jan 24;8:770264. doi: 10.3389/fnut.2021.770264 (PMC8819138; doi:10.3389/fnut.2021.770264)

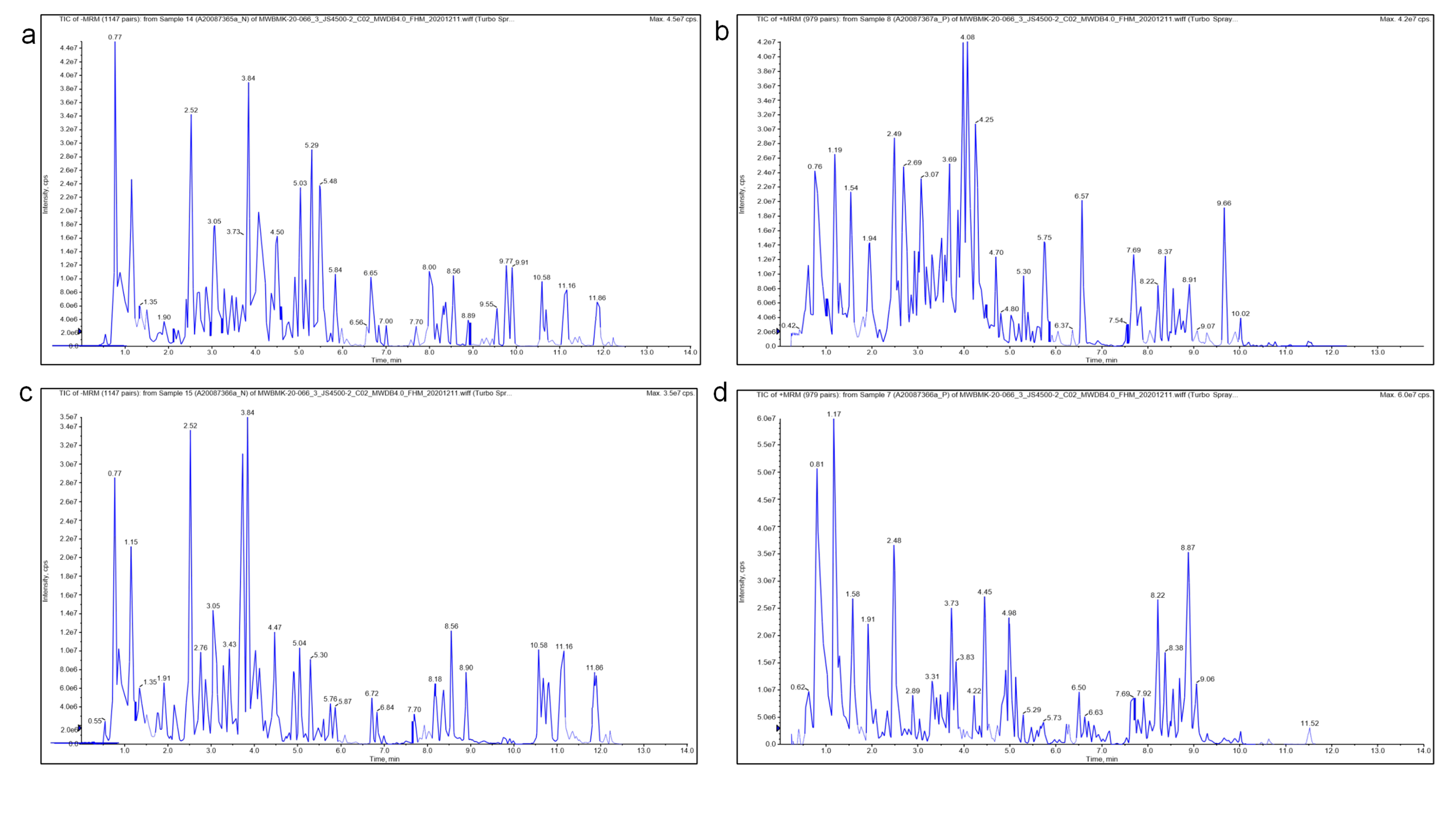

Supplement: Supplementary file 1 [file Image_1.PNG]
